# Hybrid Aquila optimizer–Harris Hawks optimization for CNN hyperparameter tuning in brain tumor classification

**DOI:** 10.1038/s41598-026-43329-7

**Published:** 2026-03-09

**Authors:** Manoj Kumar, Noor Mohd, G. Shivam, Ankur Goyal, Deepak Parashar, Rijwan Khan

**Affiliations:** 1 Graphic Era (Deemed to be University), Dehradun, India; 2 Symbiosis International Deemed University, Symbiosis Institute of Technology, Pune, Maharashtra India; 3 Manipal Institute of Technology, Manipal Academy of Higher Education, Manipal, India; 4 Marwadi University, Rajkot, Gujarat India

**Keywords:** Arithmetic Optimization Algorithm, Brain Tumor Classification, Harris Hawks Optimization, Hybrid Metaheuristic Optimization, Magnetic Resonance Imaging, Cancer, Computational biology and bioinformatics, Engineering, Mathematics and computing, Oncology

## Abstract

Magnetic resonance imaging (MRI) is hard to categorize properly in terms of interclass similarity, there is data imbalance, and sensitive clinical decision-making: but the performance of convolutional neural networks (CNNs) highly relies on effective, yet computationally costly, hyperparameter tuning. To find solutions to such issues, the given paper proposes a hybrid solution to the problems of the Aquila Optimizer and Harris Hawks Optimization, i.e., Aquila Optimizer-Harris Hawks Optimization (AO-HHO) framework, to integrate the positive qualities of extremely good global exploration of the Aquila Optimizer and the good local exploitation process of a Harris Hawks Optimization to achieve balanced and robust CNN hyperparameter optimization. On a publicly accessible dataset of 7, 023 brain MRI images divided into glioma, meningioma, pituitary tumor, and non-tumor, the proposed algorithm has been tested on with fine-tuning critical hyperparameters, such as learning rate, batch size, number of filters, dropout rate, and optimizer type. The rate of accuracy, precision, recall and F1-score of the AO-HHO-tuned CNN is invariably high than the conventional metaheuristic algorithms, including the Particle Swarm Optimization (PSO), Genetic Algorithm (GA), and Whale Optimization Algorithm (WOA) that are approximately 78–83. The proposed method also helps in reducing the cost of computing. It takes only 77.85 s to train, while the baseline optimizers take more than 300 s. This shows that AO–HHO is a reliable, accurate, and computationally efficient framework that can be used for medical imaging decision-support applications that need to be done in real time and with limited resources.

## Introduction

Accuracy and computational efficiency are critical performance criteria in modern machine learning applications, particularly as models grow increasingly complex and data-intensive. As the number of hyperparameters related to machine learning and deep learning models grows, traditional optimization methods are becoming less and less effective. Finding the best hyperparameter settings is still a time-consuming and computationally difficult task that often requires a lot of trial and error^1,2^. Bad tuning of hyperparameters can greatly lower predictive performance because they have a direct effect on model convergence, generalization capacity, and training stability^3,4^. Most machine learning models have two types of parameters: model parameters, which are learned during training, and hyperparameters, which are set by the user and affect the learning process and the model’s structure. The learning rate, the batch size, the depth of a network and regularization variables are a few of the hyperparameters that would be highly important in defining the degree to which a model will be efficient^5^. Given the learning rates in neural networks or in decision trees, say the depth of the tree, in an example, this may result in underfitting or overfitting hence this makes it more challenging to generalize. Even though they are quite common and a lot of individuals manually adjust and default library settings, these are not necessarily the most appropriate and they might rely on the dataset^6–9^. Therefore, the use of automated hyperparameter optimization (HPO) methods has proven to be an allowable substitute, since it enables a structured search of vast and high-dimensional search spaces, with lower generalization error^10^.

The automated HPO methods have taken significant interest in the metaheuristic optimization algorithms due to its flexibility, gradient free and the capability to avoid local optima. Still despite their popularity, combining them with deep learning models, most typical metaheuristics, including Genetic Algorithms (GA), Particle Swarm Optimization (PSO), and Whale Optimization Algorithm (WOA), are likely to face serious problems with the convergence speed, cross-run stability, and computational complexity in high-dimensional search spaces^11–13^. One of the most restrictive fields of these limitations is medical image analysis, such as brain tumours classification as this task can demand a reliable time-efficient model optimization in order to be used in practice in a clinical environment.

The articles on CNN-based tumour diagnosis of the brain published recently indicate that, even though the hybrid and metaheuristic optimization schemes can help improve the classification performance, they are either more computationally expensive or erratic in their convergence behavior^14–16^. This underscores the need of a framework that will be capable of optimizing diversity in global search and local refinement and deliver fast and consistent convergence in clinically sensitive procedures. In this respect, a new possible nature-based algorithm is the Aquila Optimizer (AO) that is said to be a high global exploration an algorithm, flexible in search processes and reasonable diversity and intensification. AO mimics the process of hunting the Aquila birds through numerous search processes. This enables it to easily move through high-dimensional optimization space and minimizes the chance of local minima entrapment. These properties make AO especially effective in the problem where many variables are to be optimized at the same time, e.g. the hyperparameters of a CNN. Despite the fact that AO is highly effective in global exploration, convergence of AO may be slower towards the later stages of optimization. To overcome this weakness, Harris Hawk Optimization (HGO) tool will be included in the proposed model. HHO is also reported to be quite popular in respect of its local exploitation ability, and this is because of adaptive energy-based transition systems which permit solution refining of the solutions on optimal areas to fine grains. The offered AO-HHO algorithm is the hybrid of the AO and HHO and the combination of the advantages of both optimizers, the global search of AO and the local exploitation of HHO. This hybridization leads to a higher convergence rate, a higher stability and higher optimization performance as compared to the isolated metaheuristic methods.

The given AO-HHO framework is utilized in the proposed study to CNN hyperparameter optimization to the brain tumor classification based on the MRI imaging, which is a clinically vital issue, requiring the high diagnostic performance and appropriate computational efficiency. In this environment, it is very difficult to optimize CNN hyperparameters due to interclass similarity, imbalance in data and the need to have quick and correct decision support. The primary objective of the study is to achieve higher classification accuracy and shorter training time and lowering of the computing cost with adequate adjustments of hyperparameters.

The primary findings of the paper are as follows:

- A novel hybrid AO–HHO metaheuristic optimization framework is proposed for CNN hyperparameter tuning.
- The advantages of the Aquila Optimizer are systematically leveraged and enhanced through integration with Harris Hawks Optimization to decrease the international exploration in favor of the domestic exploitation.
- The specified approach is applied to multi-class MRI brain tumor classification, which is more accurate, precise, recalls as well as F1-score than PSO, GA, and WOA.
- The validity and stability of the method developed is statistically proven in detail, multiple independent executions and significance tests.
- It can be applied in cases of resource and time constraints in medical imaging systems because it can substantially decrease the time taken to compute.
- The rest of the paper will be organized as follows: Section II will examine the literature connected to the hyperparameter optimization and CNN-based brain tumor classification. Part III provides the background of Aquila Optimizer as well as Harris Hawks Optimization algorithms. In section IV, I will outline the suggested methodology using the AO-HHO and will include data preprocessing, model architecture and optimization strategy. Section V discusses the results of the experiment and the comparison of the performance. Finally, Section VI is the conclusion of the paper and it presents a research plan in the future.

## Related work

The recent research on brain tumor classification and hyperparameter optimization has been a highly researched topic that has resulted in the development of various approaches. They suggested that it would be possible to fine-tune the hyperparameters of machine learning algorithms with the aid of four algorithms in a study by^17^ including Ant Bee Colony Algorithm, Genetic Algorithm, Whale Optimization, and Particle Swarm Optimization. The aim was to compare the computational complexity of the SVM following the hyper-tuning, whereby the Genetic Algorithm exhibited lower complexity in terms of time^18^. Computing the hyperparameters of ML algorithms requires a significant amount of computing resources. A novel approach called Opt-ABC improves convergence and accuracy by integrating artificial bee colonies, K-Means clustering, greedy algorithms and opposition-based learning. The results of the experiments show that Opt-ABC is more effective than other methods^19^ Presented a grid search-based hyperparameter tuning (GSHPT) for classifying Microarray Cancer Data using random forest parameters. The algorithm considers 10-fold cross-validation and considers optimal parameters like number of features, tree depth, and sample splits. A lot of new research has focused on classifying brain tumors with convolutional neural networks (CNNs). For example, robust MRI-based tumor classification has been presented using hybrid deep learning algorithms^20^. Additionally, effective CNN designs specifically designed for the study of brain tumors have been created^21^, and capsule networks have been studied as a substitute for recording spatial hierarchies^22^. Additionally, transfer learning has been used to classify Alzheimer’s disease in neuroimaging applications^23^, demonstrating the wider potential of CNNs in medical imaging^24^. reviewed various algorithms for fine-tuning CNN hyperparameters, including metaheuristic, statistical, sequential, and numerical approaches. It categorizes such algorithms and examines the key concepts of CNN. Comparison of HPO algorithms on various datasets is also done and their effectiveness is assessed. Furthermore^25^, employed the GAN-hyper parameter fine-tuning with the assistance of the ML and MCDM strategies through the use of Gaussian AHP. It trains GAN models with the Fashion MNIST dataset and demonstrates high advancements in the quality of generated pictures and efficiency of the computing processes. The anomalies in the data acquired under moderate cognitive impairment and Alzheimer were corrected using a hyperparameter correction method, which was developed by^26^. The technique enhances the computing effectiveness, agility, and productiveness since it simplifies AD programs, improves cross-validation execution, and shortens the time of computation by as much as 98.2. The concept of^27^ was to tune the LSTM hyperparameters to forecast stock using the Symbiotic Organism Search (SOS) metaheuristic technique. In a dataset that contained the Indonesian composite index, the hybrid SOS-LSTM model demonstrated superior performance. This approach is particularly effective for time series forecasting^28^. explored the concepts of MOO and its practical applications in machine learning^29^ turned the tuning issue into an optimization problem and offered a way to tune hyperparameters using Gaussian processes. The issue is resolved by applying Bayesian optimization, which is rooted in the Bayesian theorem. Our method successfully finds the best hyperparameters for common ML models, including neural networks and random forests, according to experiments conducted on prominent test datasets^30^ analyzed Optimizing hyperparameters for NNs on GPUs with memory constraints is made easier with Hyper Power, a framework that combines Bayesian optimization with random search. Power consumption is shown to be a low-cost constraint, and memory and power prediction methods are proposed. Hyper Power accomplishes up to 57.20 times more function evaluations while dramatically speeding up hyperparameter tuning.

The existing literature compares many strategies of hyperparameter optimization (HPO), including metaheuristic algorithms (GA, PSO, ABC), Bayesian models and hybrids, and can demonstrate higher accuracy and convergence on different areas. But the vast majority of the works pay attention to accurateness or overall performance of computing and little has been done to decrease the memory cycle time and execution time in the process of predicting heart diseases. Poor convergence rate, poor initial population and expensive cost of computation are issues that also persist especially in real time or resource constrained environments. Moreover, there are not many studies that use these methodologies on medical data exclusively in the context of cardiovascular diagnosis. This also demonstrates a gap in research in developing optimization algorithms not only more effective in optimizing models without increasing memory footprint and computation time, hence, ensuring their extrapolation to more time-sensitive or more resource-intensive machine learning tasks.

Recently, hybridization of optimization strategies and accurate CNN optimization in medical imaging and computer clouds have been studied^31–35^. Even though such techniques are effective, they usually do not have a clear convergence analysis, ablation validation and real-time feasibility. The present research is based on this literature by providing a hybrid optimizer, statistically justified, convergence analyzed and consuming less time to run and this makes AO-HHO an effective addition to the existing instruments.

## Background

The suggested hybrid solution is based on two metaheuristic algorithms; their histories are described here: Optimizers for Aquila and Harris Hawks (HHO).

### Aquila optimizer (AO)

Abualigah et al. introduced a new swarm intelligence algorithm, called AO in 2021. Aquilas have four distinct methods of hunting in which they use different types of prey. Being a hunter, an aquila may change its strategy according to the kind of preys it pursues, and use its high-speed movement and strong beak and claws to target its victim. The mathematical model can be encapsulated in the following way:

#### Expanded exploration: high soar with a vertical stoop

In this tactic, the eagle flies to a great height to investigate its surroundings, then dives vertically down once it finds its prey. This behavior is mathematically represented as:

1 $$X\left( {t+1} \right)={X_{best}}\left( t \right) \times \left( {1 - t/T} \right)+({X_M}\left( t \right) - {X_{best}}\left( t \right) \times rand)$$

2 $${X}_{M}\left(t\right)=\frac{1}{N}{\sum}_{i=0}^{N}Xi\left(t\right)$$

where X_best_(t) represents the highest point reached, X_M_(t) denotes this cycle’s average Aquilas, T represents the most iterations, with t being the current iteration, rand is an integer between zero and one, and N stands for population size.

#### Narrowed exploration: contour flight with short glide attack

This is the main tactic that Aquila uses when hunting. It descends into the chosen zone and circles the victim before attacking with quick gliding motions.

This is the formula for the position update:

3 $$X(t+1)={X_{best}}(t) \times LF(D)+{X_R}(t)+(y - x) \times rand$$

where X_R_(t) indicates the dimensional magnitude and represents the hawk’s arbitrary location. Afterwards, the Levy flying function is established: LF(D):

4 $$LF\left(D\right)=s \times \frac{u \times \sigma}{{\left|\mathrm{v}\right|}^{{\upbeta}}}$$

5 $$\sigma=\left(\frac{\varGamma(1+\beta) \times sin\left(\frac{\pi\beta}{2}\right)}{\varGamma\left(\frac{1+\beta}{2}\right) \times \beta\times {2}^{\frac{\beta-1}{2}}}\right)$$

In this case, s is set at 0.01 and β at 1.5, whereas u and v are integers that can be any number between 0 and 1. The search’s spiral configuration is shown below using the variables x and y:

6 $$\left\{\begin{array}{c}x=r \times sin\theta)\\y=r \times cos\theta\\r=r1+0.00565 \times D1\\\theta=-\omega \times D1+\frac{3\pi}{2}\end{array}\right\}$$

in which ω is set to 0.005, D_1_ variables r_1_ and D_1_ are integers that can take values between 1 and 20, where D is the dimension size and r_1_ is the number of search cycles.

#### Enhanced exploitation: low-flying strike with a gradual drop

The third step is for the eagle to dive vertically and start a preliminary attack after it has roughly located its prey. In order to sneak up on the prey and launch an assault, AO uses the chosen territory. Here is an example of this behavior:

7 $$X\left( {t+1} \right)=\left( {{X_{best}}\left( t \right) - {X_M}\left( t \right)} \right) \times \alpha - rand+\left( {\left( {UB - LB} \right) \times rand+LB} \right){\text{ }} \times \delta$$

The exploitation adjustment settings are valued by taking the top and lower limits of the issue, UB and LB, respectively of 0.1.

#### Hunting with a bow: stalking prey while walking

In this strategy, the eagle tracks its victim to its landing spot before launching a ground assault. The following mathematical expressions characterize this behavior:

8 $$X(t+1)=QF \times {X_{best}}(t) - ({G_1} \times X(t) \times rand)--{G_2} \times LF(D)+rand \times {G_1}$$

9 $$QF\left(t\right)={t}^{\frac{2 \times rand-1}{\left(1-T\right)2}}$$

10 $$\left\{\begin{array}{c}G1=2 \times rand-1\\G2=2 \times \left(1-\frac{t}{T}\right)\end{array}\right\}$$

Here we set the value of the quality function, QF(t), which was utilized to maintain the search equilibrium and we also define the current position, X(t). The avila uses a mobility parameter G1 with values between-1 and 1 to pursue prey. The flying slope (G2) drops linearly from 2 to 0 while pursuing prey.

### Harris Hawks optimization (HHO)

Recently, Heidari et al. introduced the HHO, a novel meta-heuristic optimization approach. The unusual cooperation of Harris’ hawks when hunting served as inspiration. In reaction to changes in its environment and the evasive actions of its prey, the Harris hawk displays a variety of pursuit tactics. Although the collaborative methods of the hawk of the Harris are helpful in enabling it to hunt down the identified prey until they are exhausted, putting them at a disadvantage, the oscillatory motions it makes are useful in confusing the prey trying to run away. To a nutshell, the mathematical model is the following:

#### Exploration phase

During the search of the desert, the hawks of Harris frequently make several stops. There are two perching strategies determined by the q value-dependent placements of the prey and the other members of the family.

11 $$X\left( {t + 1} \right) = \left\{ {\begin{array}{*{20}c} {X_{R} \left( t \right) - rand \times \left\| {X_{r} \left( t \right) - 2 \times rand \times X\left( t \right)} \right\|} \\ {If\;q \ge 0.5,} \\ {X_{{best}} \left( t \right) - X_{M} \left( t \right) - rand \times \left( {LB + rand \times \left( {UB - LB} \right)} \right)} \\ {if\;q < 0.5} \\ \end{array} } \right.$$

where q is a random integer ranging from 0 to 1.

#### Transition from exploration to exploitation phase

The amount of energy consumed by the prey to flee is represented by E and E0 is the initial stage of the energy. When E surpasses 1, the algorithm starts to explore, and when it is less than 0, starts exploiting.

12 $$E=2{E}_{0}\left(1-\frac{t}{T}\right)$$

Hawks use four distinct hunting methods which depend on their flying skills and their ability to track live prey. The parameter r controls both the chase mode and the amount of energy needed for flight. The victim will display this particular behavior when his chance to escape reaches 60% (*r* < 0.5) or his chance to remain at the location reaches 60% (*r* ≥ 0.5) before the attack:

(a) Soft besiege: When *r* ≥ 0.5 and |E| ≥ 0.5, Harris hawks gradually wear down their target by encircling it before launching an attack. They exploit the prey’s remaining energy, allowing it to attempt to flee, and then strategically close in to weaken it. This behavior is illustrated as follows:

13 $$X\left( {t + 1} \right) = \Delta X\left( t \right) - E\left\| {JX_{{best}} \left( t \right) - X\left( t \right)} \right\|$$

14 $$\Delta X\left( t \right) = X_{{best}} \left( t \right) - X\left( t \right)$$

15 $$J=2 \times \left( {1 - rand} \right)$$

where ΔX(t) indicates how far the prey is from its current position, and J represents the magnitude of the prey’s random jumps.

(b) *Hard besiege*: When *r* ≥ 0.5 and |E| < 0.5, Harris’ hawks encircle their prey and attack directly, as the prey is too exhausted to run away. The following positional update is applied in this setting:

16 $$X\left( {t+1} \right)={X_{best}}\left( t \right) - E{\text{ }} \times \left| {DX\left( t \right)} \right|$$

where |ΔX(t)| denotes the absolute difference between the current position of the hawk and the prey.

(c)*Soft besiege with progressive rapid dives*: When |E|≥0.5 and *r* < 0.5, in this nuanced bombardment behavior, Harris’ hawks dive repeatedly around their target while attempting to adjust their trajectory in response to the prey’s evasive movement. This behavior is represented as follows:

17 $$Y={X_{best}}\left( t \right) - E\left\| {J{X_{best}}\left( t \right) - X\left( t \right)} \right\|$$

18 $$Z=Y+S \times LF(D)$$

19 $$X\left( {t + 1} \right) = \left\{ {\begin{array}{*{20}c} {Y,} & {if\;F\left( Y \right) < F\left( {X\left( t \right)} \right)} \\ {Z,} & {if\;F\left( Z \right) < F\left( {X\left( t \right)} \right)} \\ \end{array} } \right.$$

Vector S is a stochastic variable. As for the position that comes after Y and Z, only the best one is selected.

(d) *Hard besiege with progressive rapid dives*: Because the victim doesn’t have enough energy to dodge them, full scale siege is initiated by hawks when |E| ≤ 0.5 and *r* < 0.5, in an effort to draw nearer to their prey relative to their normal placement. Violence ensues. This occurrence can be expressed mathematically as per given in Eqs. (17), (18) and (19). The next cycle will only use the best possible spot between Y and Z.

## Proposed methodology

This study presents an image classification framework that integrates an Aquila Optimizer–Harris Hawks Optimization (AO–HHO) hybrid metaheuristic for tuning Convolutional Neural Network (CNN) hyperparameters. The proposed methodology is going to involve the data preparations, model construction, hybrid optimization technique, algorithms-based workflow, the objectives formulation and evaluation which will be explainable, technically rigorous, and reproducible.

### Data collection

The present research was performed in Kaggle (brain tumor-mri-dataset) on Brain Tumor MRI Dataset (https://www.kaggle.com/datasets). It features magnetic resonance imaging (MRI) scans that is utilized to study brain tumor and is not costly to assist in carrying out reproducible research of medical imaging. Such an open dataset is applied to improve the medical image related research. Brain Tumour MRI database in question is made up of 7, 023 human brain MRI (glioma, meningoma, pituitary tumor and no tumour). The training set has 1,595 images that depict no tumor, 1,457 images that depict pituitary tumor, 1,321 images that depict glioma and 1,339 images that depict meningioma. The test sample was selected as the 405 no tumor, 300 pituitary tumor, 300 glioma and 306 meningioma pictures. The rationale behind the selection of the dataset was that it was accessible publicly, the balance was adjusted according to the classes, and lastly, it fitted the research that covered the medical image classification as long as the revisability aspect was involved.

### Data preprocessing

After collecting all the data, the second factor that will be of highest concern will be the preprocessing where all the pictures will be alike and the model will be more capable of learning. Through a proper preprocessing, the input can be normalized and noisy data can be removed and the model accuracy can be improved. The training as well as testing data were preprocessed using the same set of activities in order to make the experiment fair and repeatable.

- *Image Resizing* Image images were resized to ensure the size of all the data set were similar i.e. all the image images were resized to 64 × 64 pixels.
- *Normalization* The intensities of the pixels were normalized to the range [0,1] by dividing the values of the intensities by the value 255.
- *Data Augmentation* To improve generalization of the model and prevent overfitting, there were multiple forms of augmentation:
– Random rotation: $$\pm$$15◦.
– Horizontal flip: 50% probability.
– Zoom range: 0.1.
– Width/height shift: 0.1.
- *Label Encoding* Depending on the model requirements, class labels were encoded either as integers or using one hot encoding.
- *Data Format Conversion* All preprocessed images and labels were converted into NumPy arrays or Tensor-Flow/PyTorch tensors to ensure compatibility with deep learning frameworks.

### Model building

The evaluation of gliomas, meningiomas, pituitary tumors, and no tumors was performed using a Convolutional Neural Network (CNN) that was trained on brain MRI scans. The convolutional layers of CNNs learn spatial hierarchies of features automatically and are therefore very useful in the image-based classification tasks. We are suggesting a CNN architecture that consists of two blocks of convolutional and pooling and then two fully connected layers to achieve classification. The contents of the detailed layer are as follows:

- Conv (32) → ReLU → MaxPool.
- Conv (64) → ReLU → MaxPool.
- Flatten → Dense (128) → Dropout (0.5) → Dense (4).
- [Softmax]

This form of architecture provides the network with the capacity to isolate gradually the high level and the low-level characteristics. The dropout (0.5) layer decreases the overfitting as the neurons are randomly disabled when training and the last Softmax activation is the multiclass classification of the four types of tumors. It also had to be light in design to enable it to be optimized efficiently in addition to cost reduction computational cost.

### Optimization technique and objective function

A metaheuristic optimization solver, which is based on nature, is utilized to enhance model performance, and reduce the amount of training time. The two algorithms are used to optimize CNN hyperparameters: Aquila Optimizer (AO) and Harris Hawks Optimization (HHO). Such algorithms were selected due to complementary properties of AO where it provides good worldwide exploration and HHO efficiency of local exploitation.

The hybrid model of AO-HHO strategy will utilize AO within the initial few iterations of the optimization process to explore the global space, and HHO in the subsequent iterations to refine the good solutions and utilize the most favorable solution to accelerate the convergence and refining phases. This movement ensures that there is a moderate exploration-exploitation mechanism. The set of critical hyperparameters governing the CNN training dynamics and the predictive accuracy was optimized with the hybrid AO-HHO algorithm that would combine their merit to rule the dynamics of the CNN training. The search space that corresponds is described in Table 1.

**Table 1:** Hyperparameter search space for CNN optimization.

Hyperparameter	Search Space
Learning Rate	[0.0001–0.01]
Batch Size	16, 32, 64
Filters	32, 64, 128
Dropout Rate	0.3, 0.5
Optimizer	Adam, SGD

These parameter ranges were selected based on prior studies in deep learning and medical image classification, ensuring stable convergence and effective generalization while avoiding extreme configurations.

### Hybrid AO–HHO optimization workflow

The AO–HHO optimization process follows these steps:

i. Initialize a population of candidate solutions, where each solution represents a CNN hyperparameter configuration.
ii. Evaluate each candidate by training the CNN and computing the objective function value.
iii. Apply AO-based global exploration to diversify the search space in early iterations.
iv. Gradually transition to HHO-based local exploitation using energy-based control mechanisms.
v. Update candidate solutions iteratively until convergence criteria (e.g., change in objective function < ε or maximum iterations reached) are satisfied.
vi. Select the best-performing hyperparameter set to construct the final CNN model.

Convergence is ensured through the controlled transition from AO-driven global exploration to HHO-based local exploitation, combined with objective-function-based stopping criteria, which prevents premature convergence and promotes stable optimization.

### Objective function

The optimization objective is to minimize both classification error and training time. Let the overall objective function be defined as:

20 $$Objective\:Function:\:J\left( \theta \right) = \lambda _{1} \cdot Loss\left( \theta \right) + \lambda _{2} \cdot T\left( \theta \right)$$

Where, θ represents the learnable parameters of the CNN, T(θ) denotes the execution or training time for a given parameter set, and the weighting coefficients λ₁ and λ₂ control the trade-off between optimizing classification accuracy and maintaining computational efficiency.

*Classification Loss* Cross-entropy loss for multi-class classification is used.

21 $$\mathrm{Loss}=-\sum_{i=1}^{C}{y}_{i}\mathrm{l}\mathrm{o}\mathrm{g}\left({\widehat{y}}_{i}\right)$$

where $C$ is the number of classes, ${y}_{i}$ is the ground truth label, and ${\widehat{y}}_{i}$ is the predicted probability. The objective function guides the AO–HHO optimization to minimize classification error and training time simultaneously, using cross-entropy loss to measure prediction accuracy. This ensures the CNN achieves high classification performance while remaining computationally efficient.

### Computational complexity analysis

The computational complexity of the proposed framework can be divided into two main components: the CNN forward pass and the hybrid AO–HHO optimization. The CNN forward pass has a complexity of

22 $$\mathcal{O}(N \times {\sum}_{i=1}^{L}({L}_{i} \times {F}_{i}^{2}))$$

where $N$ represents the batch size, ${L}_{i}$ is the number of filters in the ${i}^{th}$convolutional layer, ${F}_{i}$ is the filter size for that layer, and $L$ is the total number of convolutional layers. For the AO–HHO optimization, the complexity is

23 $${\mathcal{O}}(P \times I \times T_{{CNN}} )$$

where $P$denotes the population size of candidate solutions, $I$ is the number of optimization iterations, and ${T}_{\mathrm{CNN}}$ is the training time required to evaluate a single CNN configuration. Together, these expressions capture the overall computational requirements of the proposed methodology.

### Model evaluation

For the purpose of evaluating the performance of the CNN-based classifier, several widely used evaluation metrics were employed. These metrics assess the model’s classification effectiveness and its ability to generalize across unseen data.

(1) *Accuracy* Measures the overall correctness of the model.

24 $$Accuracy=\frac{TP+TN}{FP+FN+TP+TN}$$

where TP, TN, FP, and FN represent true positives, true negatives, false positives, and false negatives, respectively.

(2) *Precision*: Indicates how many predicted positives are truly positive.

25 $$Precision=\frac{TP}{TP+FP}$$

(3) *Recall*: Indicates how well the model can identify true positive cases.

26 $$Recall=\frac{TP}{TP+FN}$$

(4) *F1-Score*: Represents the harmonic mean of precision and recall, which is particularly useful for imbalanced datasets.

27 $$F1=2 \times \frac{Precision \times Recall}{Precision+Recall}$$

All of these metrics demonstrate the classification ability of the model, which is required in such delicate applications as medical diagnostics. All the analyses are conducted on several independent analyses and the findings are provided in the form of means with +-standard deviation to obtain reliability.

## Results and discussion

This section presents a comprehensive evaluation of the proposed AO–HHO–optimized CNN model for brain tumor classification. The analysis focuses on classification performance, robustness, statistical significance, computational efficiency, and the individual contributions of the Aquila Optimizer (AO) and Harris Hawks Optimization (HHO) through a dedicated ablation study.

The proposed AO–HHO–tuned CNN was evaluated on a dataset consisting of 7,023 brain MRI images, categorized into four classes: glioma, meningioma, pituitary tumor, and no tumor. The hybrid optimizer demonstrated superior capability in exploring the hyperparameter search space and avoiding local minima, resulting in improved convergence behavior and enhanced classification performance.

### Confusion matrix analysis

**Fig. 1:**
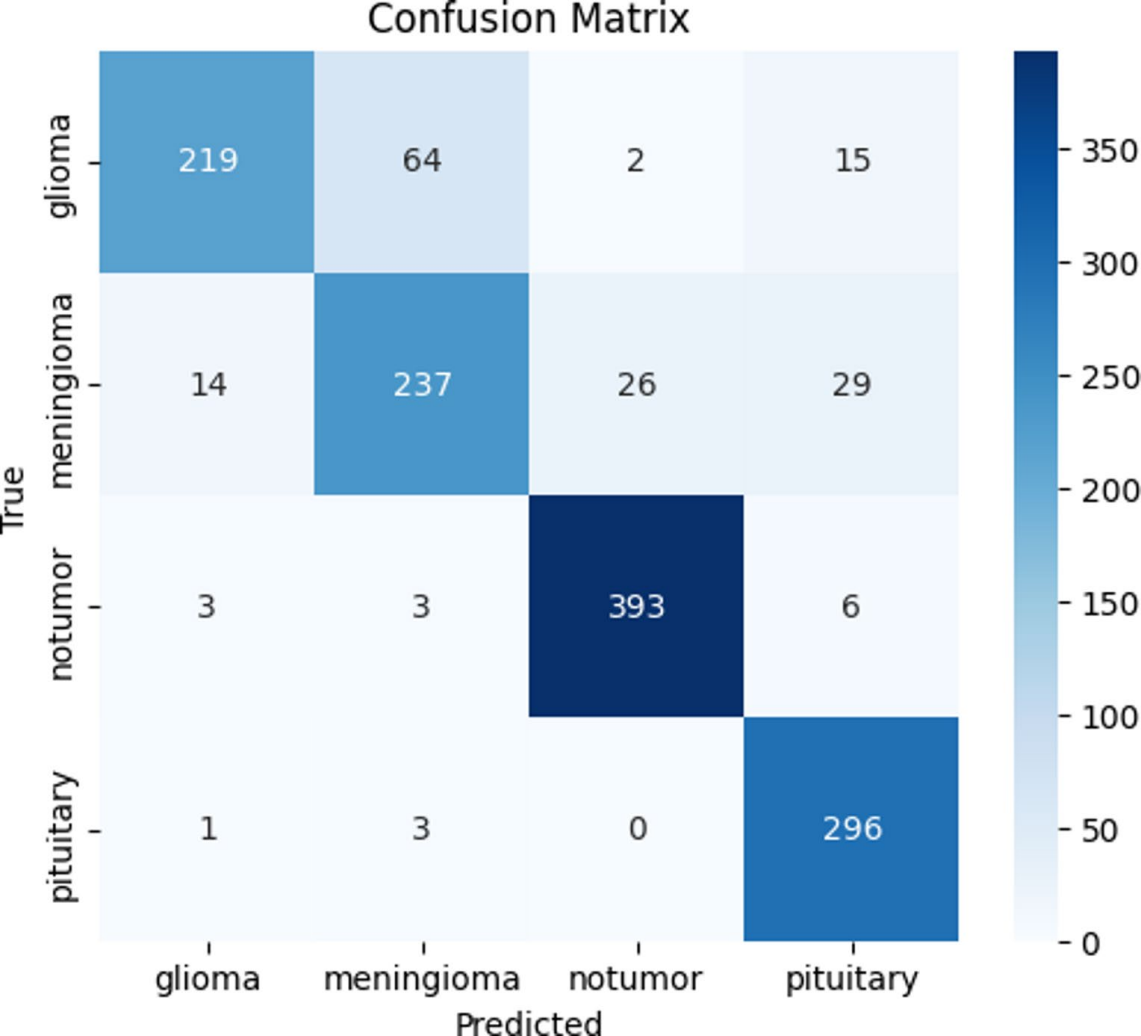
Confusion matrix of proposed AO-HHO.

**Fig. 2:**
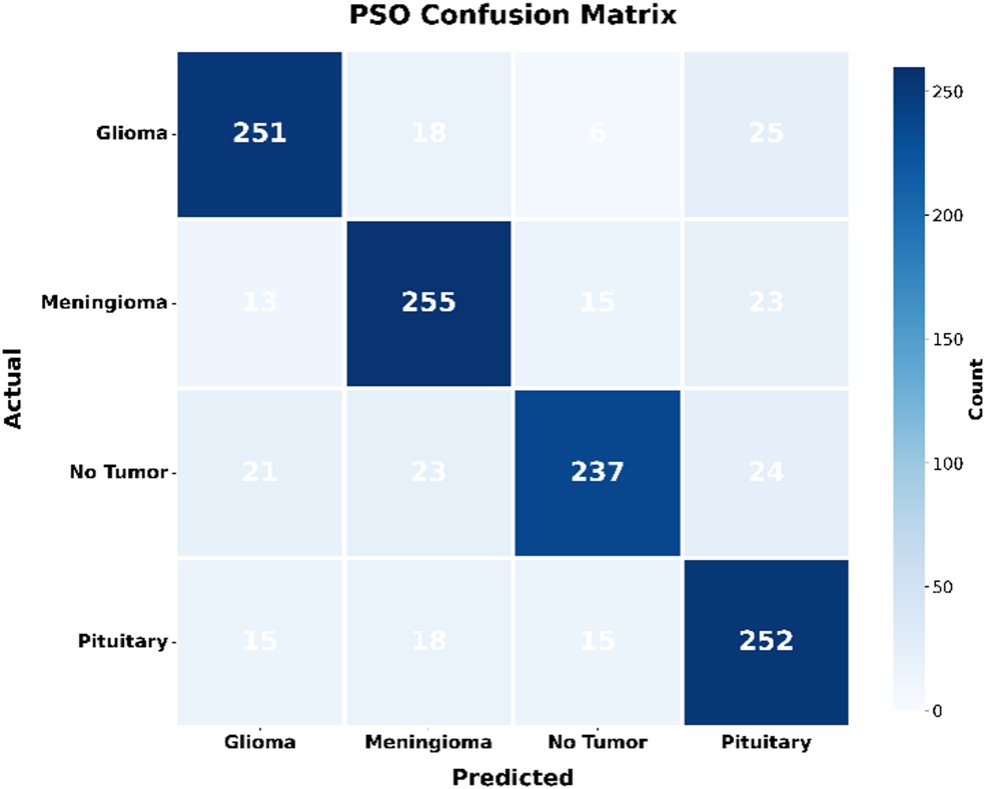
Confusion matrix of PSO.

**Fig. 3:**
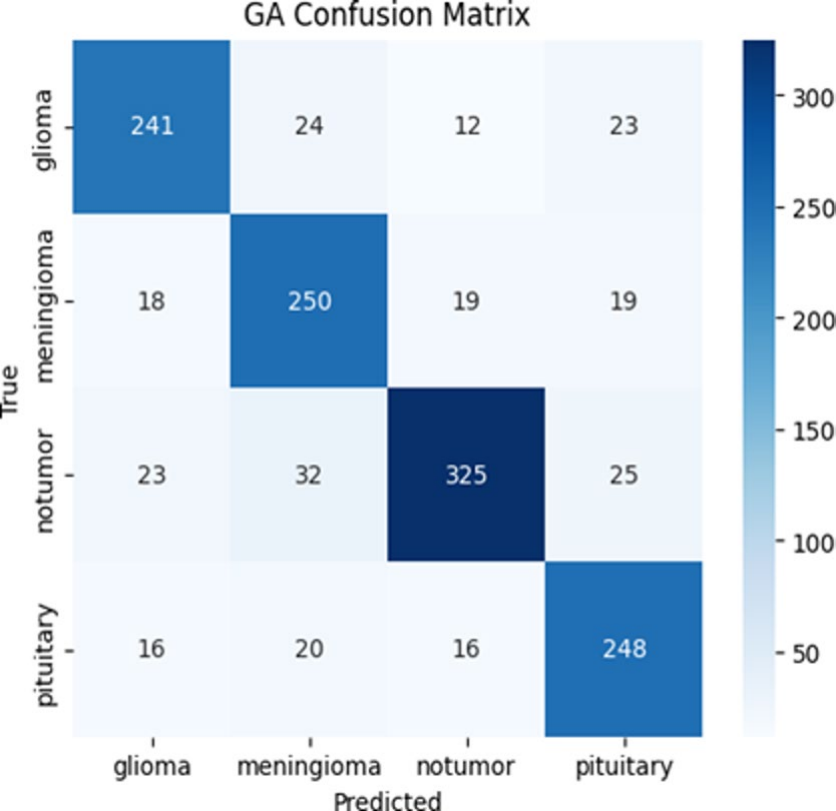
Confusion matrix of GA.

**Fig. 4:**
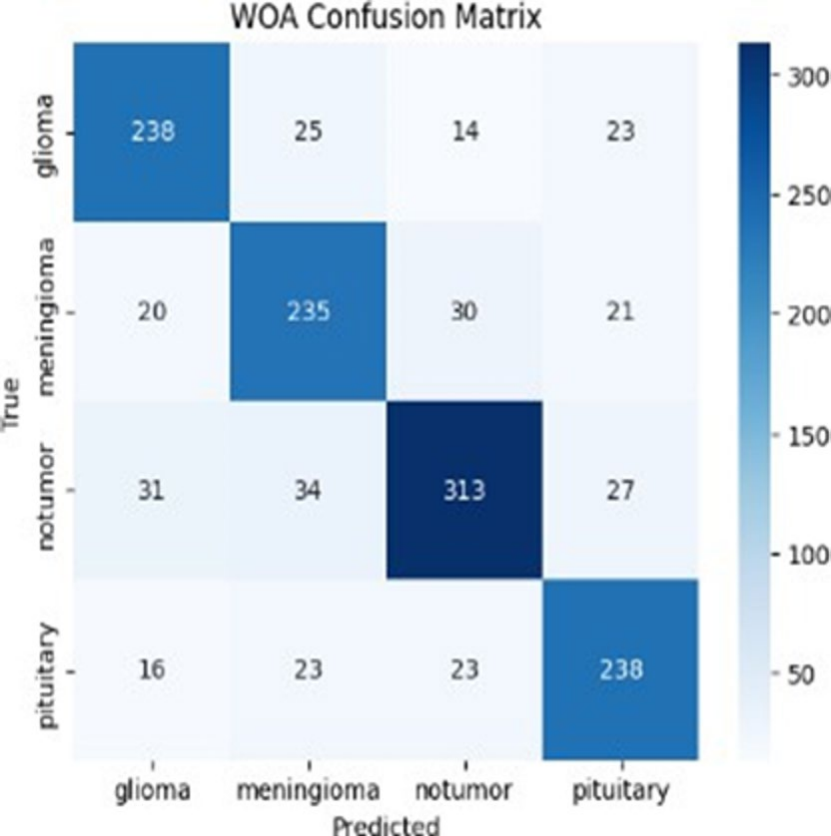
Confusion matrix of WOA.

The confusion matrix forms the visual form of the distribution of correct and incorrect predictions: The relative analysis of the four-confusion metrics highlights the improved classification performance of the proposed AO-HHO model in all types of brain tumors. As shown in Fig. 1, AO-HHO is always able to achieve high true positive values, particularly the non-tumor (393 correctly categorized) and pituitary (296 correctly categorized) classes, which have low misclassifications, and therefore the most diagonally dominant matrix among all models. On the other hand, the PSO model (Fig. 2) is an excellent performance with a slight decrease in the accurate classifications of all categories and a moderate increase in off diagonal errors especially of glioma and no-tumor. The GA model (Fig. 3) shows further decline in performance with more misclassifications in the off-diagonal cells, which indicates poor discriminative capacity. The WOA model (Fig. 4) has the highest degree of confusion of the classes and the quantity of corrects of no tumor and pituitary are much lower as compared to the other techniques, which imply low robustness. The AO-HHO model has better classification reliability and generalization capacity, and better than PSO, GA, and WOA in minimizing misclassification, and a more distinct distinction between tumor types.

### Comparative evaluation with existing models

Quantitative comparison of the proposed AO-HHO optimizer versus PSO, GA, and WOA can be seen in Table 2 with the help of accuracy, precision, recall, and F1-score as the metrics to determine the performance. The proposed AO-HHO approach is more precise as compared with PSO (83.52%), GA (81.16%), and WOA (78.11%), having accuracy of 87.34%. Equivalent improvements are also observed in accuracy, recall and F1-score. These outcomes reveal that AO-HGO improves the overall accuracy, but it remains good at balancing the false positives and the false negatives, as is significant in medical diagnosis systems. It is a superior performance of the hybrid optimizer that it can balance between global exploration and local exploitation in comparison with the traditional metaheuristic approaches. This resulted in the improvement of hyperparameter settings of the CNN.

**Table 2:** Comparative performance of optimizers.

Metric	Proposed (AO–HHO)	PSO	GA	WOA
Accuracy	0.8734	0.8352	0.8116	0.7811
Precision	0.8758	0.8325	0.8091	0.7789
Recall	0.8734	0.8355	0.8124	0.7819
F1-Score	0.8708	0.8333	0.8101	0.7801

### Statistical validation and robustness analysis

All the experiments were performed five times with various random initializations to produce reliability and reproducibility. The values of the mean and the standard deviation listed in Table 3 indicate that the AO-HHO is constantly showing better results with low variance of all the metrics, which proves the consistency of convergent conduct.

**Table 3:** Statistical validation of optimizer performance (Mean ± SD over 5 runs).

Optimizer	Accuracy (Mean ± SD)	Precision (Mean ± SD)	Recall (Mean ± SD)	F1-score (Mean ± SD)
AO-HHO (Proposed)	0.8734 ± 0.0052	0.8758 ± 0.0049	0.8734 ± 0.0061	0.8708 ± 0.0054
PSO	0.8352 ± 0.0031	0.8325 ± 0.0029	0.8355 ± 0.0030	0.8333 ± 0.0032
GA	0.8116 ± 0.0040	0.8091 ± 0.0042	0. 8124 ± 0.0039	0. 8101 ± 0.0041
WOA	0. 7811 ± 0.0035	0. 7789 ± 0.0036	0. 7819 ± 0.0037	0. 7801 ± 0.0035

**Table 4:** Pairwise statistical significance and effect size of optimizer performance.

Metric	Comparison	Mean difference	95% CI (Lower–Upper)	*p*-value
Accuracy	AO-HHO vs. PSO	0.0382	0.0251–0.0513	0.0021
	AO-HHO vs. GA	0.0618	0.0473–0.0763	0.0014
	AO-HHO vs. WOA	0.0923	0.0796–0.1050	0.0009
Precision	AO-HHO vs. PSO	0.0433	0.0290–0.0576	0.003
	AO-HHO vs. GA	0.0667	0.0522–0.0812	0.0018
	AO-HHO vs. WOA	0.0969	0.0825–0.1113	0.0012
Recall	AO-HHO vs. PSO	0.0379	0.0235–0.0523	0.0024
	AO-HHO vs. GA	0.061	0.0458–0.0762	0.0016
	AO-HHO vs. WOA	0.0915	0.0774–0.1056	0.001
F1-score	AO-HHO vs. PSO	0.0375	0.0229–0.0521	0.0027
	AO-HHO vs. GA	0.0607	0.0461–0.0753	0.0019
	AO-HHO vs. WOA	0.0907	0.0768–0.1046	0.0011

Additional statistical confirmation is given in Table 4 that tabulates the paired t-test results between AO-HHO and competitive optimizers. The statistically significant p-values (*p* < 0.05), the positive mean differences, and the narrow 95% confidence intervals all prove that performance gains that AO-HHO has obtained are not the result of random chance, but rather the result of the statistical significance. The most significant performance increases are recorded in the comparison of AO-HHO and WOA, which demonstrates the strength and efficiency of the offered hybrid strategy.

### Ablation study

An ablation analysis was conducted in order to explicitly evaluate the utility of each element of the proposed hybrid optimizer by tuning CNN hyperparameters using AO and HHO separately. The results are presented in Table 5 in a brief format.

**Table 5:** Accuracy comparison of AO, HHO, and AO–HHO.

Optimizer	Accuracy (%)
AO	83.20%
HHO	85.10%
Proposed-AO-HHO	87.34%

**Fig. 5:**
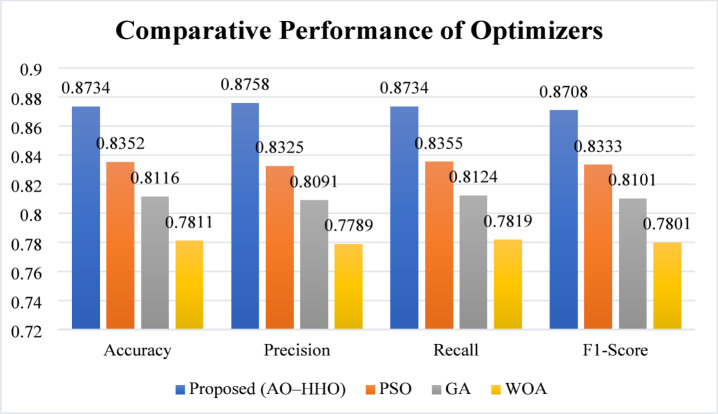
Performance comparison of optimizers.

Ao alone has an accuracy of 83.20 and this indicates that it is able to explore the world quite well and takes more time to converge when fine-tuning. The standalone HHO uses effective local exploitation to achieve a higher accuracy of 85.10% although it lacks diversity in global search. The AO-HHO hybrid, however, is the most accurate with a 87.34%. This demonstrates that using AO and HHO is a combination that is effective (Fig. 5).

In this ablation study, it is evident that AO is beneficial in maintaining the diversity of the population and promoting global exploration, whereas HHO accelerates the convergence and enhances the domestic refinement. The hybrid AO-HHO represents a decent compromise between exploration and exploitation, and thus it is superior to optimization compared to the two constituent parts. These findings are confirmation that the proposed hybrid optimizer was designed in the best way.

### Computational time analysis

**Fig. 6:**
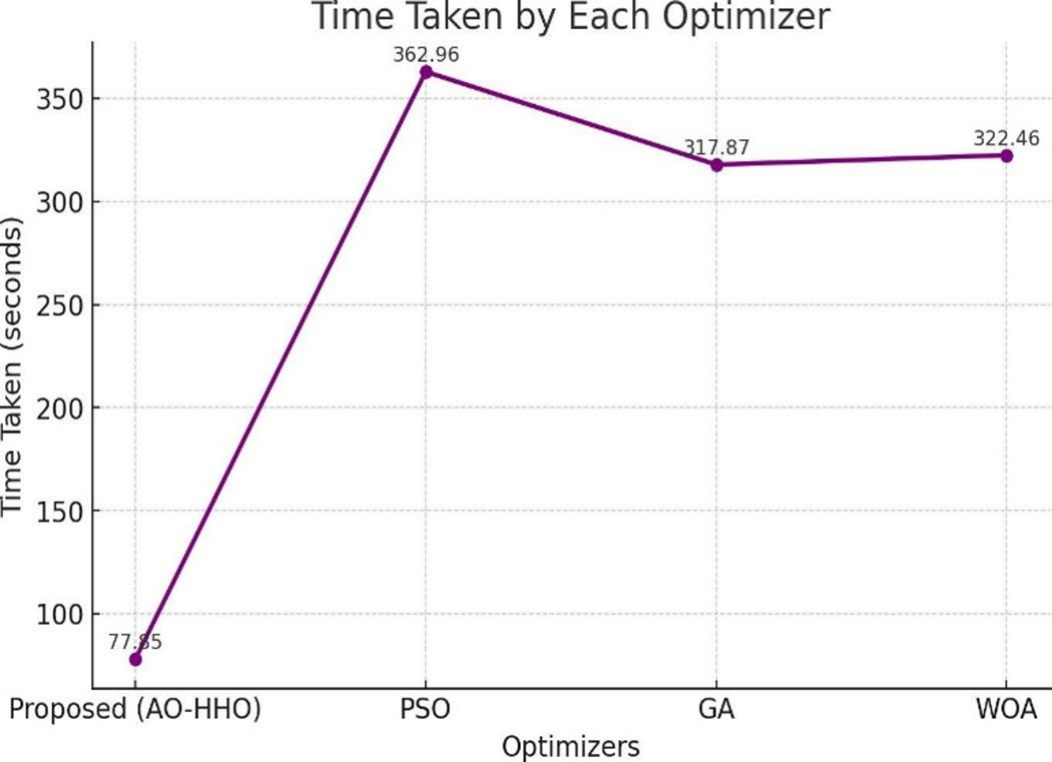
Time comparison of AO-HHO with other optimizer.

For medical applications that need to work in real time and don’t have a lot of resources, computational efficiency is very important. Table 6 shows that the proposed AO–HHO finishes training in 77.85 s, which is faster than all the other optimizers that were tested. On the other hand, PSO, GA, and WOA take 362.96 s, 317.87 s, and 322.46 s, respectively (Fig. 6).

**Table 6:** Computational time comparison of AO–HHO with other optimizers.

Optimizer	Time Taken (s)
**Proposed (AO-HHO)**	**77.85**
PSO	362.96
GA	317.87
WOA	322.46

The significant decrease in execution time shows that AO–HHO converges more quickly by quickly finding good hyperparameter settings and skipping evaluations that aren’t needed. Because of its speed and better accuracy at classifying things, AO–HHO is especially good for clinical decision-support systems.

This substantial reduction in computational time with AO–HHO, combined with its superior accuracy, demonstrates its efficiency and suitability for practical applications where both speed and performance are critical. If you are using Word, use either the Microsoft Equation Editor or the *MathType add-on* http://www.mathtype.com) for equations in your paper (Insert | Object | Create New | Microsoft Equation or MathType Equation). “Float over text” should not be selected.

On the whole, the experimental tests prove that the suggested AO-HHO-optimized CNN is in all cases more precise, robust, and computationally effective than classical metaheuristic optimization techniques. The confusion matrix analysis demonstrates that the issues of the discrimination between classes have improved, the statistical validation demonstrates that the findings can be trusted, and the ablation study demonstrates that AO and HHO are compatible partners. AO-HHO strategy balances both global exploration and local exploitation; therefore, it prevents premature convergence, and it also provides rapid and consistent optimization. Nonetheless, the suggested approach has some limitations: (i) the hybrid optimization involves additional computational costs associated with training since CNNs are repeatedly evaluated on the same dataset, which may complicate its application to other types of images and clinical conditions; (ii) the size of the population and the choice of iteration parameters influence the performance, thus, it will have to be optimized to apply to different data and clinical contexts. Despite such drawbacks, the suggested approach remains rather effective in real-time diagnostic applications and complex multi-class medical images categorization in case of appropriate computer settings.

## Conclusion

To categorize brain tumors in MRI images, this paper presents a CNN hyperparameter optimization model, which is a combination of AO and HHO. The suggested technique decreased the computation time to 77.85 s and enhanced the accuracy and precision of classification, recall, and F1-score to 87.34%. Regarding the accuracy and the efficiency of computing, AO HHO is superior to PSO, GA, and WOA. Based on the results, the suggested solution is effective in clinical cases when the decisions need to be made quickly and based on the accurate and reliable information. Future studies can use this method in other areas of medical imaging, with better deep architectures, and explore hybrid ensemble methods to enhance diagnostic results.

## Data Availability

The data that support the findings of this study are available from the corresponding author upon reasonable request.
